# Lice community structure infesting *Trinomys iheringi* (Thomas, 1911) - Ocurrence, sex bias and climatic variables on tropical island

**DOI:** 10.1016/j.ijppaw.2020.11.004

**Published:** 2020-11-18

**Authors:** Elizabete Captivo Lourenço, Ana Carolina Lacerda, Helena Godoy Bergallo

**Affiliations:** a Mammal Ecology Laboratory, Ecology Department, Rio de Janeiro State University (UERJ), Rua São Francisco Xavier, 524, Pavilhão Haroldo Lisboa da Cunha 220, Maracanã, Rio de Janeiro, RJ, 20.550-900, Brazil; b Graduate Program in Applied Ecology, Department of Ecology and Conservation, Lavras Federal University, Lavras, Minas Gerais, 37.200-900, Brazil

**Keywords:** Aggregation, Probability of occurrence, Prevalence, Humidity

## Abstract

Few studies have provided information on parasitological indexes or other ecological characteristics of lice populations that parasitize small mammals in the Neotropical region. We used lice parasitizing a rodent species, *Trinomys iheringi,* as a study model to investigate the effect of parasite occurrence and intensity on the body condition of rodents and the effect of climatic conditions, sex and age class of *T. iheringi*. We also provide information on prevalence, mean intensity, aggregation, sexual ratio of adult lice, and proportion between life stages and lice parasitizing *T. iheringi*. The study was conducted in Ilha Grande, an island in southeastern Brazil. We used a logistic regression to run a model of lice occurrence using climatic variables (rainfall, temperature, humidity), and then adding sex and age classes. A total of 39 *T. iheringi* individuals were captured with 17 parasitized (43.58%). These animals hosted *Gyropus (martini) martini* (n = 94), *Gliricola* sp*.* (n = 8), and *Pterophthirus wernecki* (n = 2). The model with humidity and sex variables showed that the occurrence of lice was negatively associated with humidity. There are more males than females infested with lice, while most of the young individuals are not infested. The higher lice occurrence in the low humidity coincides with the birth period of *T. iheringi*. The contact among individuals are higher during the reproductive period of the host species, but males are more suscetible to the lice parasitism due to higher testosterone levels that reduce the immunocompetence. The distribution pattern of lice was aggregated, but there was no correlation between body condition index and lice infestation intensity. We highlight that the major occurrence of lice occurs in the driest period of the year, that males are more prone to parasitism by lice than females, and adults more prone than young.

## Highlights

• An aggregate distribution pattern was observed for lice.
• Infestation intensity and prevalence were not related to body condition index.
• The incidence of lice is highest in drier periods.
• Males of *Trinomys iheringi* were more prone to parasitism, especially at the peak of the reproductive season.
• The proportion is two males infested per female.
• Males are the main route of transmission of lice.

## Graphical abstract

**Figure:**
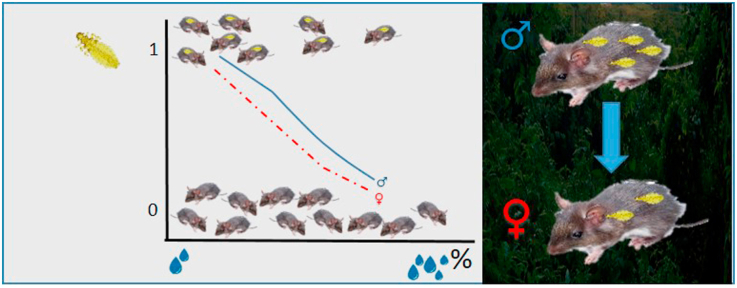

## 1 Introduction

Lice (Insecta: Phthiraptera) are highly specialized, permanent ectoparasites hosted exclusively by birds and mammals (Johnson and Clayton 2003; Ford et al., 2004; Light et al., 2010). The entire life cycle is spent on the host, which generates high specificity, with lice species restricted to one host or a group of hosts (Durden and Musser 1994a; Bittencourt 2003; Valim 2009). For this reason, lice are considered an important reference group for evolutionary studies (Linardi et al., 1991; Clayton et al., 1999; Light et al., 2010). Transmission occurs through direct physical contact between hosts while mating, due to aggressive behavior, or mother-offspring contact (Lee and Clayton 1995; Johnson and Clayton 2003).

The order Phthiraptera includes four suborders, of which Amblycera, Ischnocera, and Rhynchophthirina, are denominated chewing lice. These lice have specializations for feeding on hair, sebaceous secretions, mucus, skin, eggs or nymphs of other lice species as well as on eggs of ectoparasite mites (Ford et al., 2004). Amblycera and Ischnocera are more often found on wild birds, but also occur on mammals, while Rhynchophthirina are parasites of mammals only (Freitas et al., 2002; Johnson and Clayton 2003). The fourth suborder, Anoplura, covers sucking lice, which are exclusively mammal hematophagous ectoparasites (Marshall 1981a; Durden and Musser 1994b; Johnson and Clayton 2003). High diversity along with many adaptations to different hosts resulted in more than 3000 known species of lice (Smith 2000; Durden 2001; Johnson and Clayton 2003; Ford et al., 2004). In Brazil, 195 Amblycera species, 290 Ischinocera, 37 Anoplura and no Rhynchophthirina have been catalogued so far (Valim and Kuabara 2020).

Only some studies have provided information on parasitological indexes or other ecological characteristics of lice populations that parasitize small mammals in Brazil, and even for a Neotropical region. Population and morphometric studies were conducted on lice that occur on and are harmful to domestic or laboratory rodents such as *Cavia porcellus* (Linnaeus, 1758) (Valim et al., 2004; Pereira et al. 2012, 2013). Bittencourt and Rocha (2002) described the preferential occurrence of *Pterophthirus wernecki* Guimarães (1950), *Gyropus lineatus* Neumann, 1912 and *Gliricola (Gliricola) porcelli* (Schrank, 1781) on rodents in Ilha Grande, RJ. Fernandes et al. (2012) studied the influence of sex, body size and capture locality of the rodent *Oligoryzomys nigripes* Olfers, 1845 on lice abundance in the genus *Hoplopleura* Enderlein, 1904. Bittencourt (2003) described parasitological indexes, associations with other lice, and the sex ratio of *P. wernecki*. Other studies reported the occurrence of lice species on mammals (e.g. Guitton et al., 1986; Linardi et al., 1987; Oliveira et al., 2001; Bittencourt and Rocha, 2003; Reis et al., 2008; Valim and Linardi, 2008; Saraiva et al., 2012; Regolin et al., 2015; Sponchiado et al., 2015), including the description of several species (e.g. Werneck 1934; 1935; 1936; 1942; 1948; Timm and Price 1999).

There are many lice species associated with the order Rodentia, from chewing lice of the suborder Amblycera to sucking lice of the suborder Anoplura (e.g. Castro and Gonzalez, 1997, Kim 1988; Werneck 1948; Soliman et al., 2001; Bittencourt 2003; Valim 2009; Light et al., 2010; Archer et al., 2014). One rodent that hosts lice, *Trinomys iheringi* (Thomas, 1911) (family Echimyidae) is distributed from the south of São Paulo state to the south of Rio de Janeiro state (Attias et al., 2009). Species of this genus are nocturnal with terrestrial habitats, and have a diversified diet including fruits, herbs and grains (Paglia et al., 2012; Sena and Lessa 2020). Four lice species have already been reported on *T. iheringi* on Ilha Grande, on the southeastern coast of Rio de Janeiro state: *Trimenopon jenningsi* (Kellogg & Paine, 1910), *P. wernecki*, *Gl. porcelli* and *Gy. lineatus* (Guitton et al., 1986; Bittencourt & Rocha 2002, 2003). Conducting new studies on Ilha Grande, we used lice parasitizing *T. iheringi* as a study model to investigate the relationship of parasite occurrence and intensity on the body condition of rodents as well as the relationship of climatic conditions, sex, and age class of *T. iheringi* on lice infestation. We also provide information on parasitological indexes, aggregation, sexual ratio of adult lice, and proportion between life stages and lice parasitizing *T. iheringi*.

We established three hypotheses: (i) the body condition of rodents is correlated with the occurrence and parasitic load of lice, thus hosts with lice have lower body condition index. This occurs due to the harm caused by the infestation on the health of the host or by the sick hosts not being able to groom and this increase the infestation of lice, which can be measured by the body condition index (Fitze et al., 2004; Hawlena et al., 2006; Lucan 2006; Schwensow et al., 2011; Sánchez et al., 2018); (ii) the highest intensity of lice occurrence occurs during the lowest period of rainfall during the reproductive peak of *T. iheringi* (Bergallo 1995; Patterson et al., 2015), and (iii) male rodents are more heavily infested due to the higher likelihood of contact with other individuals in the population and the reduced immunocompetence due to higher testosterone levels (Bergallo 1994, 1995; Clayton and Tompkins 1994; Hillgarth 1996; Zuk and McKean 1996; Ferrari et al., 2004; Fernandes et al., 2012; Patterson et al., 2015).

## 2 Material and methods

**Fig. 1:**
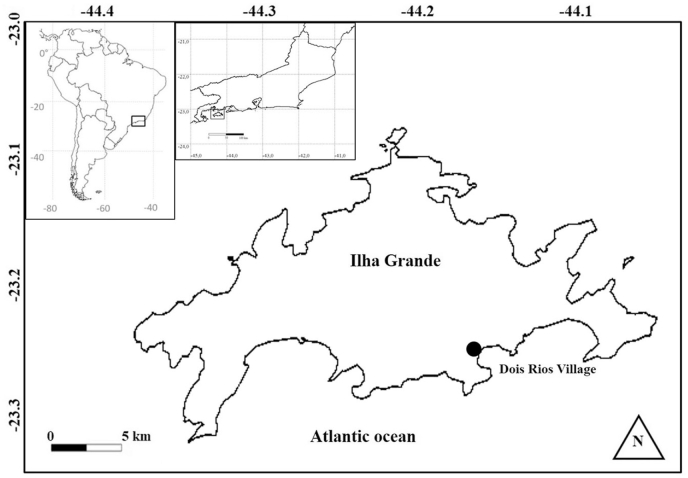
Location of capture of *Trinomys iheringi* in Dois Rios Village, Ilha Grande, Rio de Janeiro State, Brazil, between April 2013, and December 2015.

This study was conducted in Ilha Grande State Park, situated in the municipality of Angra dos Reis, Rio de Janeiro state, southeastern Brazil (Fig. 1). The island covers approximately 193 km^2^, the shortest distance to the continent being about 2 km (Araújo and Oliveira 1988). The isolation of Ilha Grande from the continent began about 7000 years ago (INEA - Instituto Estadual do Ambiente, 2013). The vegetation on the island is represented by Atlantic Forest (Dense Ombrophilus Forest in the Brazilian Classification System), restinga (coastal scrub), and mangroves (INEA - Instituto Estadual do Ambiente, 2013). The climate is humid tropical, classified as Af by Köppen, with rainfall higher than 2,242 mm, rainfall distributed throughout the year with higher intensity in the summer and lower in winter. July is the coldest month (average 20.2 °C) and February is the hottest month (average 26.4 °C) (INEA - Instituto Estadual do Ambiente, 2013).

Small rodents were captured for our study in the surroundings of Dois Rios village, near the Parnaioca (−23.182861°, −44.193611°) and Caxadaço (−23.176908°, −44.185921°) trails between April 2013, and December 2015, in 20 capture campaigns over 60 nights. The survey was conducted on 18 sampling grids. Each grid covered 1600 m^2^, and grids were at least 200 m apart. Nine traps (Sherman or Tomahawk) were set in each area along three 40 m long transects in parallel lines 20 m apart. The soil traps were thus 20 m apart (at distance points zero, 20 and 40 m). One trap was also set in the undergrowth between 1.5 m and 2.5 m above the ground, and another one on a tree, close to the canopy, at heights between 4.5 m and 12 m. The traps were baited with bananas, deployed during three consecutive nights, and checked every morning. The small mammals captured were weighed, marked with ear tags for individual identification (Powell and Proulx 2003), measured, had the sex identified (Brasil 2002), the ectoparasites removed, and were then freed at the same point of capture.

To collect ectoparasites, the entire body of each rodent was brushed with a toothbrush over a jar containing 70% ethanol. We brushed until we no longer find ectoparasites by visual search. The time invested in this procedure per animal was not recorded. After brushing, the liquid was either transferred directly to a flask, or a paper filter was used to sieve the ectoparasites, then the filter with the ectoparasites was stored in an identified flask with 70% ethanol. The brush was set aside and no longer used before it could be properly cleaned. The lice were diaphanized in 90% lattic acid and mounted between lamina and laminula in Hoyer's medium (Krantz and Walter 2009) in the Mammal Ecology Lab of the Rio de Janeiro State University (UERJ). Seven dichotomous keys and descriptions available from the scientific literature were used for species identification (Werneck 1942, 1948; Guimarães 1950; Johnson 1972; Emerson and Price 1975; Price et al., 2003; Valim 2009).

The indexes of prevalence, average abundance, average infestation intensity (Bush et al., 1997), and discrepancy (Poulin 1993) were analyzed in the Quantitative Parasitology 1.0.14 software (Reiczigel et al., 2019). The discrepancy index (D) calculates parasite aggregation in the host population and varies between 0, when all hosts have the same number of parasites, to 1, when all parasites are found on one single host individual (Poulin 1993). The chi-square test was performed to verify possible differences between the sexual proportions of adults and life stage (adult and nymph) of the lice collected. Host recaptures were excluded.

The residual of the linear regression between the natural logarithm of weight (In W) and the natural logarithm of head and body length (In HB) was used as body condition index (Jakob et al., 1996; Bergallo and Magnusson 2002). We used a covariance analysis (ANCOVA) on the size of the rodents (ln HB) as covariate to test the difference in body condition among individuals infected and uninfected by lice. We also used the Spearman (Rs) correlation between parasitic load and body condition index to examine the correlation of parasites on the health of each animal.

A logistic regression was run to model louse occurrence using climatic variables (total rainfall, average temperature, and average humidity of the months when captures were conducted). We then added sex and age classes to the best logistic regression model to evaluate the likelihood of lice occurrence. *Trinomys iheringi* individuals were classified as young or adult according to body mass. Animals with W ≤ 190 g were considered young females, and animals with W ≤ 180 g, young males (Bergallo 1995). Analyses were performed in Systat software version 13.

Meteorological data were obtained from the automatic weather station of the National Institute of Meteorology, Paraty Station, Rio de Janeiro state, about 55 km away from the study area.

## 3 Results

A total of 39 *T. iheringi* individuals were captured, 17 of which hosted parasites (43.58%). These animals hosted 104 lice specimens in three species: *Gyropus (martini) martini* (Werneck, 1948), *Gliricola* sp*.*, and *Pterophthirus wernecki* (Table 1). Thirteen animals hosted only *G. (m.) martini* individuals. One adult male (W = 330 g, HB = 21 cm) hosted one *G. (m.) martini* specimen and one *Gliricola* sp*.* specimen, while another adult male (W = 243 g, HB = 20 cm) hosted three *G. (m.) martini* specimens and seven *Gliricola* sp. specimens (Supplementary material 1).

**Table 1:** Lice parasite indexes, abundance, and ratio between males and females, adults and nymphs, and nymphal stages on *T. iheringi* in Ilha Grande State Park, Rio de Janeiro state, Brazil. *p < 0.05

	*Gyropus (martini) martini*	*Gliricola* sp.	*Pterophthirus wernecki*	Total
Total abundance	94	8	2	104
Prevalence (%)	41,00 (25,60-57,90)	5,00 (0,60-17,30)	3,00 (1,00–13,50)	43,58 (27,80-60,40)
Average intensity	5,88 (2,19-16,40)	4,00 (1,00–4,00)	2,00	6,12 (2,76-15,00)
Average abundance	2,41 (0,95-6,95)	0,21 (0,00-0,77)	0,05 (0,00-0,14)	2,67 (1,13-7,05)
Discrepancy index (D)	0,84 (0,76-0,92)	0,94 (0,87-0,95)	0,95 (0,85-0,95)	0,87 (0,75-0,91)
Male/female abundance	8/17	2/3	1/1	11/21
Male/female proportion	1 : 2,12	1 : 1,5	1 : 1	1 : 1,91
Adult/nymph abundance	25/69 *	5/3	2/0	32/72*
Adult/nymph proportion	1 : 2,76	1,67 : 1	2:0	1 : 2,25
Abundance nymph I/II/III	38/18/13	–	–	–
Proportion nymph I/II/III	2,85: 2,05:1	–	–	–

The population of *G. (m.) martini* was composed by more nymphs than adults (69 nymphs/25 adults; 2.76:1) (χ^2^ = 26.833, p = 0.03), with 17 females, 8 males, 38 nymphs in instar I, 18 nymphs in instar II, and 13 nymphs in instar III. Of the 16 *T. iheringi* parasitized by *G. (m.) martini*, 14 had a parasitic load of one to four lice. An adult male (W = 185 g, HB = 18 cm) captured in December 2014, hosted 21 *G. (m.) martini* specimens, of which three were adult males, nine adult females (1:3), and nine nymphs.

**Fig. 2:**
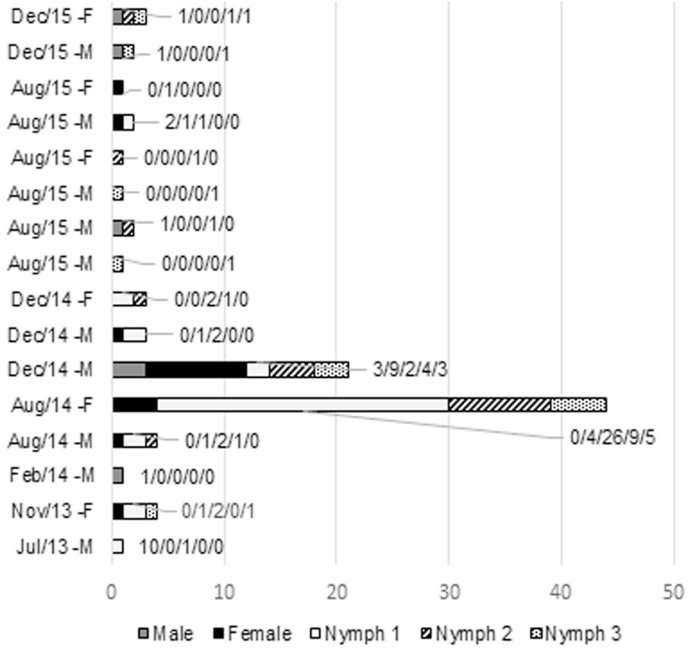
Distribution of *Gyropus (m.) martini* stages on *Trinomys iheringi* rodents. Host sex (F = female, M = male) and capture months (Aug = August, Dec = December, Feb = February, Jul = July, Nov = November) in Ilha Grande State Park, RJ, Brazil. The numbers along the x axis represent the number of lice life stages: male/female/nymph 1/nymph 2/nymph 3.

The host with 44 specimens was an adult female (W = 275 g, HB = 19.5 cm) captured in August 2012, hosting only four females and 40 nymphs in different instars (26 nymphs I/9 nymphs II/5 nymphs III) (Fig. 2). The distribution frequency of *G. (m.) martini* indicates an aggregate pattern (Table 1). There were three *Grilicola* sp. females, two males, and three nymphs (5 adults/3 nymphs; 1.67:1), and only one *P. wernecki* female and one male on a host (Table 1).

**Fig. 3:**
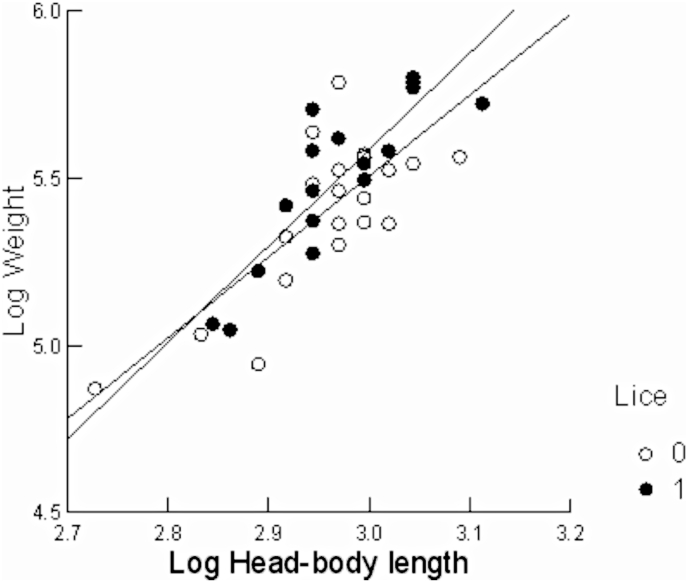
Relationship between the natural logarithm of body mass and the natural logarithm of body length for *Trinomys iheringi* individuals infected and uninfected by lice in Ilha Grande State Park, RJ, Brazil. Open circles and the continuous line refer to uninfected individuals, while solid circles and the dashed line refer to infected individuals.

The relationship between the natural logarithm of mass and the natural logarithm of head-and-body length was significant (r = 0.812; F = 64.092; P < 0.001, N = 39), but no difference was observed between infected and uninfected individuals, according to the no significance on the slope (F = 0.495, P = 0.486) and intercept (F = 1.842, P = 0.183) (Fig. 3). There was also no correlation between body condition index and parasitic load (Rs = 0.178, p = 0.286, N = 38).

**Fig. 4:**
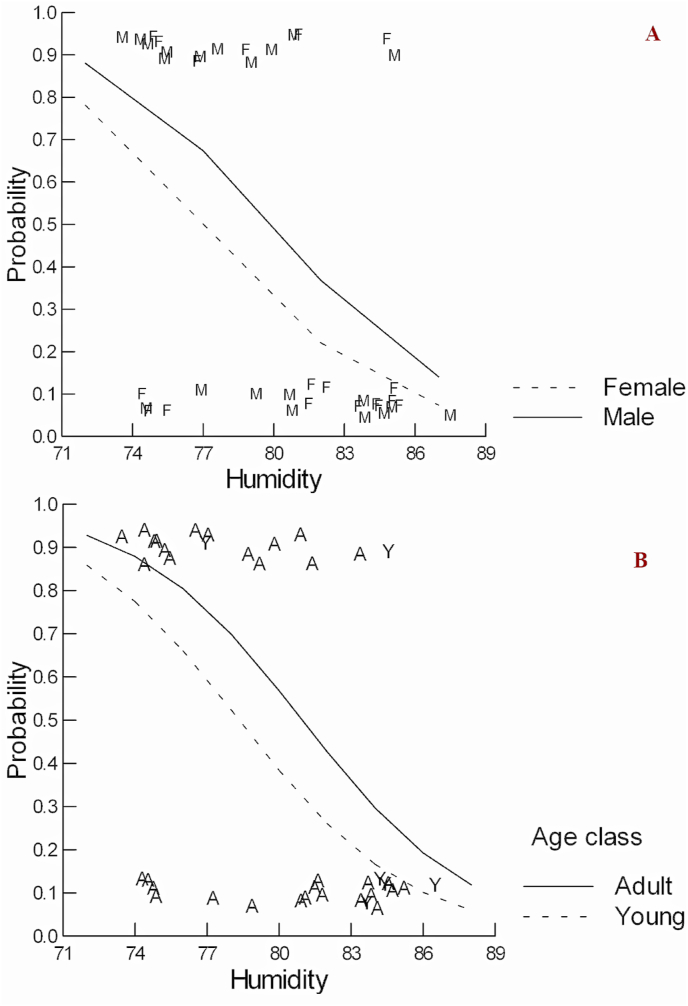
Probability of lice occurrence on *Trinomys iheringi* as a function of humidity and sex (A), and humidity and age class (B) in Ilha Grande State Park, RJ, Brazil. The letters in the upper part of the graph represent the presence of lice on rodents, and the letters in the lower part of the graph indicate the absence of lice on rodents.

The climatic variable that best explained the likelihood of occurrence of lice was humidity (Table 2). The model with humidity and sex variables (ꭕ^2^ = 9.461; p = 0.0088) showed that the occurrence of lice was negatively associated with humidity (odds ratio = 0.776 ± 0.076; p = 0.0094). Sex did not significantly affect lice occurrence, but the ratio was approximately two males infected to one female infected (odds ratio = 0.486 ± 0.360) (Fig. 4A). The function that explained lice occurrence on *T. iheringi* in this model is y = exp (20.237–0.253*Humidity-0.721 × Sex)/[1+ exp (20.237–0.253*Humidity-0.721 × Sex)]. The model which used humidity and age class (young or adult) was also significant (ꭕ^2^ = 8.923; p = 0.015), and came second in higher log-likelihood value (Table 2). The occurrence of lice was negatively associated with humidity (odds ratio = 0.752 ± 0.081; p = 0.008), and although age class did not significantly affect lice occurrence, more adults than young from *T. iheringi* were infected (odds ratio = 0.473 ± 0.543) (Fig. 4B). The function that explains lice occurrence on *T. iheringi* is y = exp (23.040–0.285*Humidity-0.748 × AgeClass)/[1+ exp (23.040–0.285*Humidity-0.748 × AgeClass)]. Hence, there are more males than females infested with lice, while most of the young individuals are not infested (Fig. 4).

**Table 2:** Regression logistic model to describe lice occurrence on *T. iheringi* hosts in Ilha Grande State Park, Brazil. The best models with the highest log-likelihood used humidity and sex, followed by humidity and age class.

Parameter	Estimate	Standard error	Z	p-Value	Model
CONSTANT	−20.2371	7.7695	−2.6047	0.0092	ꭕ^2^ = 9.4606 **p** = **0.0088** Log-Likelihood = −21.9810
Humidity	0.2534	0.0976	2.5962	**0.0094**	
Sex	0.7213	0.7413	0.9729	0.3306	
CONSTANT	23.0399	9.0046	2.5587	0.0105	ꭕ^2^ = 8.9229 **p** = **0.0115** Log-Likelihood = −22.2499
Humidity	−0.2846	0.1073	−2.6526	**0.0080**	
Age class	−0.7483	1.1467	−0.6526	0.5140	
CONSTANT	−22.5244	11.0674	−2.0352	0.0418	ꭕ^2^ = 8.5887, **p** = **0.0136** Log-Likelihood = −22.4170
Humidity	0.2886	0.1453	1.9861	**0.0470**	
Precipitation	−0.0020	0.0069	−0.2946	0.7683	
CONSTANT	−20.2837	7.7114	−2.6304	0.0085	ꭕ^2^ = 8.4998, **p** = **0.0036** Log-Likelihood = −22.4615
Humidity	0.2580	0.0970	2.6589	**0.0078**	
CONSTANT	−0.4761	0.5245	−0.9077	0.3640	ꭕ^2^ = 3.3032, p = 0.0691 Log-Likelihood = −25.0597
Precipitation	0.0079	0.0045	1.7527	0.0796	
CONSTANT	0.7266	3.0580	0.2376	0.8122	ꭕ^2^ = 0.0238, p = 0.8774 Log-Likelihood = −26.6994
Temperature	−0.0208	0.1349	−0.1542	0.8774

## 4 Discussion

Population structure and descriptive data of three lice species associated with *T. iheringi* are presented in this study in relation to climatic conditions and host conditions. Although insular fauna are generally characterized by a reduced number of species the lice fauna parasitizing one single species of rodent, *T. iheringi*, on Ilha Grande seems to have a high richness, comparing with other studies in islands (Magnanou and Morand 2006; Regolin et al., 2015), totaling at least five species after completion of this study, which added one species (Guitton et al., 1986; Bittencourt & Rocha 2002, 2003). Several studies conducted on Ilha Grande contributed to the knowledge of the high richness of parasites on the island (Guitton et al., 1986; Bittencourt & Rocha 2002, 2003). Bittencourt’ studies (2003, Bittencourt and Rocha 2002, 2003) were carried out in the same region of this study (Dois Rios Village), although with a smaller sampling effort (12 months, 7474 night-traps) and recorded three lice species. Guitton et al. (1986) present a list of species, with four species to *T. iheringi* for the Praia Vermelha region, 16 km away from the collection area of this study and in the continental side of the island, with sampling of 1976–1977.

Although this is the first record of *Gyropus (martini) martini* at Ilha Grande, it was the species of highest abundance*.* This species of louse uses *Trinomys dimidiatus* (Günther, 1877) as type host in the Corcovado type locality in the municipality of Rio de Janeiro, but is also present on the same host species in records of other areas in the municipality of Angra dos Reis (Werneck 1934, 1948; Valim and Linardi, 2008).

*Grilicola* sp. is morphologically similar to *Grilicola porcelli* (Schrank, 1781), but has not yet been described (unpublished data). It is possible that the report on *G. porcelli* on Ilha Grande refers, in fact, to this new species (Guitton et al., 1986, Bittencourt and Rocha, 2003). *Grilicola porcelli* is a species associated with the genus *Cavia* Pallas, 1766 (Caviidae) (Werneck 1948; Regolin et al., 2015), but there are several records of occurrence on other species of different Rodentia families (Werneck 1948). This species is widely distributed in Brazil and also occurs in the United States, Peru, Paraguay, Venezuela, and England (Ewing 1924; Werneck 1948; Emerson and Price 1975; Linardi et al. 1987, 1991). Emerson and Price (1975) found *Cavia porcellus* (Linnaeus, 1758) individuals hosting up to 84 *G. porcelli* specimens in Venezuela (84, 80, 44, 35 and others in smaller numbers), while in this study we observed only eight specimens of *Grilicola* sp. on *T. iheringi.* More detailed taxonomic revisions of *G. porcelli* specimens reported for Ilha Grande are required to confirm their identification.

Rodents in the suborder Hystricomorpha (Caviidae and Echimyidae) are usual hosts to *Pterophthirus, Gliricola* and *Gyropus* species (Vanzolini and Guimarães 1955, Durden and Musser, 1994a a; b). The majority of Gyropidae species is associated with Caviidae and Echimyidae (Price and Graham 1997). *Proechimys*, *Thrichomys* and *Trinomys* are hosts to half of the 27 species of *Gyropus* (Valim and Linardi, 2008). Bittencourt (2003) reports high infestation of *P. wernecki* on *T. iheringi* on Ilha Grande, with 453 specimens on 19 hosts (54.3% prevalence, 12.9 of mean abundance and 23.8 ± 52.9 of mean intensity) and 234 specimens on one single individual. Only two adults of *P. wernecki* were found on *T. iheringi* in this study, which was conducted in the same area ten years later. On the other hand, Bossi et al. (2002) did not collect any species of lice in 75 capture efforts conducted over a year on *T. iheringi* in the Juréia-Itatins Ecological Station, São Paulo state, Brazil, despite using the same capture methods. Bittencourt (2003) observed an association of *P. wernecki* with *Gy. lineatus* and *Gr. porcelli*, but we were unable to find any association between *P. wernecki* with other lice species. The records of *P. wernecki* are still exclusive to Ilha Grande and its type locality in Boraceia, São Paulo state, both on *T. iheringi* (Guimarães 1950; Bittencourt & Rocha 2002, 2003; Bittencourt 2003).

We found a larger quantity of female than male lice on *T. iheringi*. This follows the general bias, as the sexual ratio of adult lice shows higher female abundance (e.g. Marshall 1981b; Clayton et al., 1992; Gupta et al., 2007; Pereira et al., 2012; Archer et al., 2014). This bias is apparently related to several factors, such as longevity, infrapopulation size, prevalence, and environmental factors (Marshall 1981a,b, Pap et al., 2013, Archier et al. 2014). Besides, female bias can be explained because males are more active and smaller than females and have copulation-modified legs that may reduce their ability to cling to the host (Marshall 1981b; Pap et al., 2013). Therefore, they are more likely to be separated from the host body, being thus more susceptible to predation by the host or to being killed by adverse climatic or nutritional conditions (Marshall 1981b; Pap et al., 2013).

The proportion of adults and nymphs may provide some clues on the temporal stability of lice populations (Marshall 1981b; Gupta et al., 2007). According to Marshall (1981a,b), the presence of more adults may indicate population decline, while the presence of more nymphs would indicate population growth. However, variations in age structure in lice populations, as well as in ectoparasites, occur over time (Marshall, 1981a a; b). Higher numbers of nymphs in the first instar may be considered common, as mortality increases with development (Maturano and Daemon 2014). Bittencourt (2003) found more *P. wernecki* nymphs than adults, while we found only two adults, of which the female was fertilized, with eggs visible inside the body.

The distribution pattern of ectoparasite populations is usually aggregated (e.g., Lee and Clayton 1995; Clayton et al., 1999; Archer et al., 2014; Padam et al., 2015). In this study, 42.31% of 104 lice specimens lived on one single host. However, even on some more heavily infested individuals, no relationship was found between parasitic load and body condition. Therefore, our hypothesis that the condition of *T. iheringi* individuals infected by lice would differ from that of uninfected rodents was not corroborated. The correlation between parasites (occurrence and infestation intensity) and body condition indexes vary in different parasite-host systems, but high parasite infestations tend to negatively affect body condition (e.g. Hawlena et al., 2006; Sánchez et al., 2018; Eads 2019). It is possible, however, that high parasitic loads may have effects that have not been measured in this study (see Fitze et al., 2004; Hawlena et al., 2006; Sánchez et al., 2018). High parasite infestations cause harm to domestic and wild animals, as they may cause itching, alopecia, irritation, or scarification of the skin, resulting in severe injuries aggravated by secondary infections that may lead to weight loss or reduced productivity (Bildfell et al., 2004; Esteruelas et al., 2016; Taylor et al., 2016; Eads 2019).

High aggregation may be a distribution pattern of lice species that are not often in contact with other hosts, so transmission occurs in the reproductive period of the host, during copulation (Clayton and Tompkins 1994; Hillgarth 1996; Zohdy et al., 2012). Aggregation is reduced in host species that live in groups, as the chance of horizontal transmission is higher (Lee and Clayton 1995; Rózsa 1997). Some species in the Echimyidae family share some degree of sociability. The life territories of males and females of *Proechimys semispinosus* Tome, 1860, for example, are overlapped, and the nests are shared for a short period of time (Seamon and Adler 1999; Endries and Adler 2005). Higher sociability has been observed in *Trinomys yonenagae* (Rocha, 1995), with spatial overlap of adults at burrow entrances (72%) and sharing of burrows among individuals (67%) (Santos and Lacey 2011). However, this does not seem to be the case of *T. iheringi,* as male life territories are larger and overlap with males and two or three females over the reproductive period, while female life territories do not overlap (Bergallo 1994, 1995). High lice aggregation may indicate lower sociability by *T. iheringi* and thus higher horizontal transmission only during the reproductive period, with males as the main transmitters of lice, since they are promiscuous and can have contact with several females (Bergallo & Magnusson 1999, 2004).

The hypothesis that the largest lice infestation would take place in the period of lower rainfall was partially correct. The climatic variable that best explained the presence of lice in the logistic regression was humidity, not rainfall. However, periods of higher rainfall correspond to higher humidity in the region (r = 0.67, p=<0.001, data in Supplementary Material 1). This may not indicate a direct correlation with abiotic conditions, but an indirect effect on the host due to climatic conditions (Stanko et al., 2015; Patterson et al., 2015). Still, some species of either sucking or chewing lice parasitizing mammals or birds are abundant even when humidity is low, but there may also be high abundance in high humidity (>90%) (Matthysse 1946; Murray 1957; Marshall, 1981b; Santos et al., 2006). Matthysse (1946) reports that the best environmental conditions for the incubation of *Bovicola bovis* (Linnaeus, 1758) (=*Damalina bovis*) parasitizing cattle occurs when humidity is between 70 and 84% (35 °C), egg eclosion is delayed four days when humidity is between 10 and 50% (35 °C), and no eclosion occurs when relative humidity reaches 90%. Murray (1957) reported that *Bovicola ovis* (Linnaeus, 1758) (=*Damalinia ovis*) also prefers lower humidity for oviposition on sheep and that high humidity has a depressive effect on populations. Santos et al. (2006) indicate that *Bovicola caprae* (Gurlt, 1843) populations are sensitive to humidity, occurring in reduced numbers when humidity increases as a consequence of high rainfall. They also state that the nymph stage is more sensitive to humidity, precipitation and temperature. Although temperature and solar radiation that can increase the temperature in skin and increase the lice mortality, are the abiotic factors more closely associated with lice oviposition and survival (Murray 1968), we were unable to observe a correlation with temperature. We assume that it is because *T. iheringi* is mainly a night forest species with low exposure to sunlight and because the temperature in the study area has a small variance on average, from 20.2 during the coldest month to 26.4 in the hottest month of the year.

Reproduction of some ectoparasite species may be synchronized with the reproductive period of the host species, facilitating dispersal (e.g. Marshall 1981b; Christe et al., 2000; Patterson et al., 2015). The chances of contact between individuals are higher during the reproductive period of the host species, especially for males in polygynous mating systems (Durden 1983; Zuk and McKean 1996; Fernandes et al., 2012; Zohdy et al., 2012; Patterson et al., 2015). *Trinomys iheringi* can reproduce throughout the year, but a higher birth proportion was observed in the driest period in Jureia-Itatins Ecological Station, São Paulo state, Brazil (Bergallo 1994, 1995). This bias of more parasitized males, mainly in the reproductive period, has already been reported for other parasite-host relationships (Fernandes et al., 2012; Patterson et al., 2015). Abundance of fleas on red squirrels (*Tamiasciurus hudsonicus* (Erxleben, 1777)) was significantly higher in males in the mating period than post-mating period, in Canada (Patterson et al., 2015). Sucking lice (*Lemurpediculus verruculosus* (Ward, 1951)) transfers peaked during the breeding season of lemurs (*Microcebus rufus* (É. Geoffroy, 1834)) due to increased social interactions between hosts in Madagascar (Zohdy et al., 2012). The transfer of the sucking lice *Hoplopleura erratica* (Osborn, 1896) was most prevalent during the mating period, presumably because of increased of chipmunks (*Tamias striatus* (Linnaeus, 1758)) contacts in USA (Durden, 1983). Furthermore, with the increase of social interactions during the reproductive season, males experience increases in testosterone, that is an immunosuppressant, and consequently are more vulnerable to parasitic infestation (Zuk and McKean 1996; Klein 2000). During the wet periods, most young *T. iheringi* were free of lice. We can assume that males transfer lice to females during the reproductive period (horizontal transmission), then females pass them on to their offspring (vertical transmission).

The lice fauna observed on *Trinomys* at Ilha Grande by Guitton et al. (1986) were *Gy. lineatus*, *Gl. porcelli, T. jenningsi* and Hoplopleuridae individuals; Bittencourt and Rocha (2002, 2003) added *P. wernecki* (perhaps the same Hoplopleuridae species reported by Guitton et al., 1986), and this study added *Gy.(m.) martini* as well as a new species of *Gliricola* which is currently being described. The distribution pattern of lice was aggregated, but there was no correlation between body condition index and lice infestation intensity. We highlight that the major occurrence of lice occurs in the driest period of the year, when males are more prone to parasitism by lice than females, and adults more prone than young. Although we suggest that males are the main vector of lice transmission among rodents, studies on the dynamics of lice transmission and on the perpetuation of lice infrapopulations in young rodents (female or male) need to be verified, as well as the possibility of lice being removed from offspring due to parental care (grooming).

## Declaration of competing interest

The authors declare there are no conflict of interest.
